# A retrospective analysis of incident pregnancy in phase 1 and 2a HIV-1 vaccine study participants does not support concern for adverse pregnancy or birth outcomes

**DOI:** 10.1186/s12879-021-06431-x

**Published:** 2021-08-11

**Authors:** Lynda Stranix-Chibanda, Chenchen Yu, Margaret Brewinski Isaacs, Mary Allen, Jessica Andriesen, Stephen R. Walsh

**Affiliations:** 1 University of Zimbabwe, Harare, Zimbabwe; 2 Vaccine and Infectious Disease Division, Fred Hutchinson Cancer Research Center, Seattle, WA USA; 3 Division of AIDS, National Institute of Allergy and Infectious Diseases, Bethesda, MD USA; 4 Division of Infectious Diseases, Brigham and Women’s Hospital, Boston, MA USA; 5 Harvard Medical School, Boston, MA USA

**Keywords:** HIV vaccine, Safety, Immunogenicity, Maternal-child health, Pregnancy

## Abstract

**Background:**

Pregnancies occur during HIV-1 vaccine clinical trials, despite requirements for women of reproductive potential to use effective contraception. Deployment of an effective HIV-1 vaccine regimen will likely target adolescents and young adults and therefore safety for pregnant and breastfeeding women will need to be addressed.

**Methods:**

We performed a retrospective, cross-protocol analysis to identify and compare pregnancy outcomes reported in 53 Phase 1 and Phase 2a HIV-1 vaccine clinical trials conducted by the HIV Vaccine Trials Network (HVTN).

**Results:**

Two thousand six hundred seventy-three women of reproductive potential were identified and 193 pregnancies were reported. 39 of 53 (74%) studies had at least one pregnancy reported with an overall pregnancy rate of 3.15 per 100 woman-years (w-yr). While active contraception use was required during study participation, 13 of the 53 studies also contained a long-term follow up period during which pregnancy was no longer discouraged. The pregnancy rate during main study participation was 3.09 per 100 w-yr, while pregnancies occurred at a slightly greater rate in the long-term follow up period (3.22 per 100 w-yr). Adverse pregnancy outcomes were reported at similar rates between vaccinees and placebo recipients when vaccine vectors, adjuvant used, or geographic region were examined.

**Conclusion:**

Although there is considerable heterogeneity amongst the different vaccine trials, there appears to be no obvious indication of increased risk of adverse pregnancy or birth outcomes in these early phase HIV-1 vaccine studies. More complete data on pregnancy outcomes should be collected in early phase HIV-1 vaccine clinical trials to better inform subsequent efficacy trials.

**Supplementary Information:**

The online version contains supplementary material available at 10.1186/s12879-021-06431-x.

## Introduction

Some significant progress has been made this decade to develop a safe and effective vaccine that protects against HIV infection. UNAIDS estimates there were 1,600,000 new adult HIV infections in 2018, the majority in those aged 15–24 years, with young women in Sub-Saharan Africa bearing the brunt of the epidemic [1]. Future deployment of an effective HIV vaccine will likely target adolescents and youth in an effort to provide vaccine-elicited protection prior to the onset of sexual activity. To ensure durable protection throughout high-risk periods, booster vaccination may also be necessary.

For females of reproductive potential, sex that could lead to HIV infection may also result in pregnancy. Conversely, the perinatal period is a time of heightened risk for new HIV infection [2–5]. Additionally, acute HIV infection during pregnancy or while breastfeeding carries a 10-fold increased risk of transmission to the child [6, 7]. Aligning with the UNAIDS ethical guidance pertaining to HIV vaccine trials [8], women are purposely included to obtain safety, immunogenicity, and efficacy data for vaccine candidates in both sexes. However, pregnancy is actively discouraged in vaccine trial participants because of unknown safety implications for the fetus. Investigators therefore exclude pregnant and breastfeeding women from participating and require women of reproductive potential to use effective contraception throughout the vaccination phase of the trial. These restrictions may deter women from enrolling in HIV vaccine trials or lead to discontinuation of vaccination prior to receipt of the full regimen or early study attrition for those who do enroll [9, 10]. Pregnancy testing is conducted regularly for females of reproductive potential who enroll in a vaccine trial, including prior to every scheduled vaccination. If a participant becomes pregnant, study vaccine administration is discontinued and she is encouraged to remain in follow-up for ongoing safety monitoring and to periodically collect systematic pregnancy and birth outcome data in person or via e-mail or telephone. Some protocols may allow resumption of the vaccination schedule after a participant is no longer pregnant. Hence, intensive contraception counselling is performed with women of reproductive potential and on-site provision of contraception services is a preferred approach in many settings [11].

Since its formation in 2000, the HIV Vaccine Trials Network (HVTN) has opened more than 80 vaccine trials with over 20,000 volunteers enrolled [12]. Study participants are typically healthy individuals 18 through 50 years of age, with increasing numbers of females of reproductive potential being involved. On-study pregnancies do occur despite pregnancy being discouraged and contraception being required, albeit rarely in early-phase trials. The requirement to report pregnancy outcomes provides some limited data regarding the incidence of pregnancy under these conditions as well as pregnancy and birth outcomes amongst study participants. As a result, a large data set is available with which to conduct a cross-sectional analysis of pregnancy outcomes in recipients of a diverse group of study vaccines compared with placebo. In this paper, we present a retrospective analysis of incident pregnancies which occurred during early phase (Phase 1 or 2a) HIV vaccine studies conducted by the HVTN.

## Methods

This retrospective, cross-protocol analysis included all pregnancies reported during Phase 1 and Phase 2a HVTN vaccine clinical trials, including during long-term follow-up (LTFU) periods. Studies that did not involve active vaccination with an HIV vaccine were excluded, and only those studies for which participants and study staff were unblinded to treatment assignment prior to December 31, 2018 were included. Efficacy (Phase 2b or Phase 3) studies, some of which only enrolled participants born male, were excluded from this analysis. Of note, though efficacy studies are not included in this analysis, the HVTN 503 efficacy study data have previously been published [11].

Study participants were healthy volunteers without HIV-1 infection ages 18 through 50 years who were at low risk for acquiring HIV as per standard criteria [13]. The individual study protocols were approved by the institutional review boards and, when applicable, biosafety committees at all sites and written informed consent was obtained from each participant.

Pregnancy report and outcome data gathered from study Case Report Forms (CRFs) were tabulated from 53 phase 1 and 2a HIV vaccine trials conducted by the HVTN between 2002 and 2018. These trials were conducted in multiple countries, though the majority of participants were from the United States. Pregnancies were reported from time of enrolment through final study visit. The vaccination phase varied by study from a single injection up to 36 months.

Additionally, 13 studies also contained a long-term follow up (LTFU) period after the vaccination phase was completed for continued safety monitoring during the next 28 to 42 months during which pregnancies were also reported. To normalize across studies and different study phases, pregnancy rates per 100 woman-years (w-yr) of study participation were calculated.

Female participants of reproductive potential were identified according to the individual study criteria. This included being assigned female sex at birth, and not having reached menopause (no menses for 1 year) or having undergone a hysterectomy, bilateral oophorectomy or tubal ligation. All reported pregnancies were verified by clinical research site staff using urine or serum testing.

The date of the participant’s last menstrual period (LMP) was obtained from CRF data. In the case where only the month and year were reported, a date of the 15th of the known month was used as an estimate. Additionally, if an LMP was not reported, it was estimated using the outcome date and reported gestational age at outcome. Similarly, if an outcome was reported without an outcome date, the outcome date was estimated from the LMP and the reported gestational age. All pregnancies had either an LMP or outcome date reported.

Sites were encouraged to attempt to obtain outcome information from pregnant participants as soon as possible, even after the participants’ study participation had concluded. As most studies were blinded until after the final scheduled visit by all participants had occurred, outcomes reported during the active phase of studies were generally collected in a blinded fashion. Pregnancy outcome data, where known, were categorized as follows: therapeutic/elective abortion (as reported on the CRF), spontaneous abortion (< 20 weeks gestation), spontaneous fetal demise and/or stillbirth (≥20 weeks), premature live birth (< 37 weeks), or full-term live birth (≥37 weeks). Outcomes of spontaneous abortion, spontaneous fetal demise and/or stillbirth, and premature live birth were considered to be adverse outcomes. Adverse events of moderate or greater severity, including congenital anomalies, occurring within a window of 7 days prior to the pregnancy outcome and 56 days following the outcome were also compiled and tabulated from CRF data.

Treatment assignment information (receipt of study product or placebo) was classified according to type of product administered (viral vector, protein/peptide, or DNA), viral vector type and adjuvant. Participants were considered in receipt of product if they received at least one study product administration. Placebo recipients were grouped for analysis. Participants were also separated according to whether vaccinations were discontinued due to the identification of the incident pregnancy or whether the full course of vaccinations was completed prior to the identification.

Participants born female who were not considered of reproductive potential according to the protocol in which they participated were excluded. Pregnancies reported via standardized CRFs were compiled and tabulated. The total number of participants reporting being assigned female sex at birth, of reproductive potential and having received at least one dose of an experimental HIV vaccine or placebo served as the denominator. Descriptive statistics were used to summarize the participant characteristics, pregnancy rates, and outcome data. The associations between pregnancy outcomes and study factors, including treatment assignment, product type, adjuvant, injection status, and participant geographic region, were evaluated using Fisher’s exact test [14].

## Results

### Studies included

We reviewed data collected during 53 completed HVTN HIV-1 vaccine Phase 1 and 2a clinical trials conducted from March 27, 2001 and unblinded by December 31, 2018. Table 1 presents study characteristics, including the vaccine types (viral vectors, protein/peptide, DNA) used in the studies in which pregnancies are reported. Additional Table 1 presents characteristics of all 53 studies reviewed.

**Table 1:** Study Characteristics and Pregnancy Rates

Study	Study Treatment	Participating Countries ^**1**^	N females of reproductive potential	N participants reporting one or more pregnancies	Pregnancies (main study)	Pregnancies (Long-term followup)	Pregnancies (total)	rate main	rate ltfu	rate total	Study Reference
HIVNET 026	canarypox + protein	BR, HT, PE, TT	60	6	7	0	7	8.05	n/a	8.05	http://www.ncbi.nlm.nih.gov/pubmed/17693888
HVTN 039	canarypox	US	30	0	0	0	0	0.00	n/a	0.00	http://www.ncbi.nlm.nih.gov/pubmed/16136469
HVTN 040	VEE	ZA, BW, US	17	2	2	0	2	13.33	n/a	13.33	http://www.ncbi.nlm.nih.gov/pubmed/22914365
HVTN 041	protein	US	22	0	0	0	0	0.00	n/a	0.00	http://www.ncbi.nlm.nih.gov/pubmed/17049679
HVTN 042	canarypox + lipopeptide	US	73	7	8	0	8	4.49	n/a	4.49	http://www.ncbi.nlm.nih.gov/pubmed/25253665
HVTN 044	DNA	US	23	1	1	0	1	2.70	n/a	2.70	http://www.ncbi.nlm.nih.gov/pubmed/21940420
HVTN 045	DNA	US	20	2	2	0	2	10.00	n/a	10.00	http://www.ncbi.nlm.nih.gov/pubmed/16831092
HVTN 048	DNA	BW, US	15	3	3	0	3	14.29	n/a	14.29	http://www.ncbi.nlm.nih.gov/pubmed/18055072
HVTN 049	DNA + protein	US	45	2	2	0	2	3.77	n/a	3.77	http://www.ncbi.nlm.nih.gov/pubmed/21451004
HVTN 050	Ad5	BR, DR, HT, MW, PE, PR, ZA, TH, US	162	20	5	21	26	3.14	3.48	3.41	http://www.ncbi.nlm.nih.gov/pubmed/20854108
HVTN 052	DNA	US	76	1	1	0	1	1.61	n/a	1.61	NCT00071851
HVTN 054	Ad5	US	21	0	0	0	0	0.00	n/a	0.00	http://www.ncbi.nlm.nih.gov/pubmed/21048953
HVTN 055	FPV + MVA	BR, US	74	2	2	0	2	2.60	n/a	2.60	http://www.ncbi.nlm.nih.gov/pubmed/21216311
HVTN 056	peptide	US	35	1	1	0	1	2.27	n/a	2.27	http://www.ncbi.nlm.nih.gov/pubmed/18996425
HVTN 057	DNA + Ad5 boost	US	30	0	0	0	0	0.00	n/a	0.00	NCT00091416
HVTN 059	VEE	BW, ZA, US	37	3	3	0	3	8.57	n/a	8.57	http://www.ncbi.nlm.nih.gov/pubmed/22914365
HVTN 060	DNA + IL12 DNA + peptide	TH, US	69	3	3	0	3	4.11	n/a	4.11	http://www.ncbi.nlm.nih.gov/pubmed/22242162
HVTN 063	DNA + IL12 DNA + IL15 DNA	BR, US	53	0	0	0	0	0.00	n/a	0.00	http://www.ncbi.nlm.nih.gov/pubmed/22242162
HVTN 064	peptide	US	28	1	1	0	1	3.70	n/a	3.70	http://www.ncbi.nlm.nih.gov/pubmed/19786145
HVTN 065	DNA + MVA	US	69	2	2	0	2	2.86	n/a	2.86	http://www.ncbi.nlm.nih.gov/pubmed/21282192
HVTN 067	DNA + MVA	US	15	0	0	0	0	0.00	n/a	0.00	http://www.ncbi.nlm.nih.gov/pubmed/22398243
HVTN 068	DNA + Ad5	US	30	0	0	0	0	0.00	n/a	0.00	http://www.ncbi.nlm.nih.gov/pubmed/26908875
HVTN 069	DNA + Ad5	PE, US	37	5	1	5	6	2.70	6.17	5.08	http://www.ncbi.nlm.nih.gov/pubmed/21931737
HVTN 070	DNA + IL12 DNA	US	59	4	4	0	4	7.27	n/a	7.27	http://www.ncbi.nlm.nih.gov/pubmed/23840043
HVTN 071	Ad5	US	21	1	1	0	1	4.76	0.00	1.09	http://www.ncbi.nlm.nih.gov/pubmed/23151505
HVTN 072	DNA + Ad5 + Ad35	US	4	0	0	0	0	0.00	0.00	0.00	NCT00472719
HVTN 073/E	DNA + MVA + protein	ZA, US	22	2	3	0	3	5.45	0.00	5.00	http://www.ncbi.nlm.nih.gov/pubmed/27098021
HVTN 076	DNA + Ad5	US	6	0	0	0	0	0.00	0.00	0.00	NCT00955006
HVTN 077	DNA + Ad5 + Ad35	US	81	5	1	6	7	1.30	2.10	1.93	http://www.ncbi.nlm.nih.gov/pubmed/26587311
HVTN 078	attenuated vaccinia + Ad5	CH	57	1	1	0	1	1.45	n/a	1.45	http://www.ncbi.nlm.nih.gov/pubmed/25271627
HVTN 080	DNA + IL12 DNA	US	27	0	0	0	0	0.00	n/a	0.00	http://www.ncbi.nlm.nih.gov/pubmed/23840043
HVTN 082	DNA + Ad5	US	8	0	0	0	0	0.00	0.00	0.00	NCT01054872
HVTN 083	Ad5	US	78	4	1	4	5	1.75	1.20	1.28	http://www.ncbi.nlm.nih.gov/pubmed/26475930
HVTN 084	Ad5	BR, PE, CH, US	82	16	0	19	19	0.00	5.18	4.70	http://www.ncbi.nlm.nih.gov/pubmed/31748227
HVTN 085	Ad5	US	37	4	0	4	4	0.00	2.35	2.11	NCT01479296
HVTN 086	DNA + MVA + protein	ZA	95	9	2	7	9	2.17	3.32	2.97	http://www.ncbi.nlm.nih.gov/pubmed/27583368
HVTN 087	DNA + IL12 DNA + VSV	US	35	1	0	1	1	0.00	3.85	1.49	https://www.ncbi.nlm.nih.gov/pubmed/30235286
HVTN 088	protein	US	13	2	2	0	2	7.41	n/a	7.41	https://www.ncbi.nlm.nih.gov/pubmed/30615119
HVTN 090	VSV	US	29	5	0	6	6	0.00	7.89	6.45	http://www.ncbi.nlm.nih.gov/pubmed/26199949
HVTN 092	DNA + attenuated vaccinia	CH, US	76	2	2	0	2	3.64	0.00	3.03	NCT01783977
HVTN 094	DNA + MVA	US	30	3	1	2	3	2.27	6.45	4.00	https://www.ncbi.nlm.nih.gov/pubmed/28727817
HVTN 096	DNA + attenuated vaccinia + protein	CH	49	10	3	9	12	4.35	3.95	4.04	https://www.ncbi.nlm.nih.gov/pubmed/31601541
HVTN 097	canarypox + protein	ZA	49	2	2	0	2	3.85	n/a	3.85	https://www.ncbi.nlm.nih.gov/pubmed/31534016
HVTN 098	DNA + IL12 DNA	US	40	1	1	0	1	1.75	n/a	1.75	NCT02431767
HVTN 100	canarypox + protein	ZA	109	4	4	0	4	1.82	n/a	1.82	https://www.ncbi.nlm.nih.gov/pubmed/29898870
HVTN 105	DNA + protein	US	48	0	0	0	0	0.00	n/a	0.00	https://www.ncbi.nlm.nih.gov/pubmed/31566579
HVTN 106	DNA + MVA	CH, US	44	2	2	0	2	4.08	n/a	4.08	NCT02296541
HVTN 110	Ad4 + protein	US	4	0	0	0	0	0.00	n/a	0.00	NCT02771730
HVTN 111	DNA + protein	ZA, TZ, ZM	65	1	1	0	1	1.59	n/a	1.59	https://www.ncbi.nlm.nih.gov/pubmed/31900486
HVTN 112	DNA + VSV	US	5	0	0	0	0	0.00	n/a	0.00	NCT02654080
HVTN 203	canarypox + protein	US	81	4	5	0	5	4.24	n/a	4.24	http://www.ncbi.nlm.nih.gov/pubmed/17106277
HVTN 204	DNA + Ad5	BR, HT, JM, ZA, US	254	18	18	0	18	3.01	n/a	3.01	http://www.ncbi.nlm.nih.gov/pubmed/21857901
HVTN 205	DNA + MVA	PE, US	124	10	5	6	11	4.17	2.80	3.29	http://www.ncbi.nlm.nih.gov/pubmed/24403557
Total			2673	172	103	90	193	3.09	3.22	3.15	

^1^ – ISO 3166 codes for countries and territories: *BR* Brazil, *BW* Botswana, *CH* Switzerland, *DO* Dominican Republic, *HT* Haïti, *JM* Jamaica, *PE* Peru, *PR* Puerto Rico, *MW* Malawi, *TH* Thailand, *TT* Trinidad and Tobago, *TZ* Tanzania, *US* United States, *ZA* South Africa, *ZM* Zambia

### Contraception approach

The two earliest studies (HIVNET 026 and HVTN 203) required that site investigators reviewed “adequate birth control methods” with the participants and described specific types in study-specific ancillary documents. The 51 protocols that followed used a standard protocol template that specified a list of effective barrier, hormonal, and surgical contraceptive methods that were to be reviewed and discussed with all participants who were of reproductive potential prior to enrolment. In addition, 3 of these required that participants in South Africa agree to use two effective methods of contraception. Contraception use was required throughout the study in 47/53 studies (89%), even after the active vaccination phase was completed. Contraception use was required only during the active phase of vaccination for the remaining six studies. All studies required that sites perform pregnancy tests on the day of vaccine receipt and that negative results were obtained prior to study product administration. During LTFU, no contraception was required as pregnancy was no longer discouraged, but pregnancies and outcomes were still reported. While contraception use was required in all trials, those data are not presented in this analysis; the most recent contraception use and/or cessation of contraceptive usage was not collected on pregnancy-related CRFs.

### Maternal demographic characteristics

The studies included in this analysis enrolled 2673 females identified as being of reproductive potential in 15 countries (Table 2). At screening, median age was 26 years and BMI 24.7.

**Table 2:** Demographics of Women of Reproductive Potential

Total participants of reproductive potential		2673		
Country		N	%	
Botswana		8	0.30%	
Brazil		62	2.30%	
Dominican Republic		8	0.30%	
Haiti		27	1.00%	
Jamaica		18	0.70%	
Malawi		1	0.00%	
Peru		81	3.00%	
Puerto Rico		13	0.50%	
South Africa		471	18%	
Switzerland		158	5.90%	
Tanzania		15	0.60%	
Thailand		56	2.10%	
Trinidad and Tobago		19	0.70%	
US		1726	65%	
Zambia		10	0.40%	
Ethnicity		N	%	
Hispanic or Latino		282	11%	
Not Hispanic or Latino		2251	84%	
Unknown		140	5.20%	
Race		N	%	
White		1446	54%	
Black/African American		854	32%	
Asian/Pacific Islander		105	3.90%	
Native American/Alaskan Native		12	0.40%	
Hawaiian/Pacific Islander		4	0.10%	
Multiracial		100	3.70%	
Other or Unknown		152	5.70%	
Age at enrollment		N	%	
18–20		407	15%	
21–30		1455	54%	
31–40		496	19%	
41–50		309	12%	
50+		6	0.20%	
Median	26			
Mean	28.3			
BMI		N	%	
Underweight (< 18.5)		50	1.90%	
Normal weight (18.5–24.9)		1096	41%	
Overweight (25–29.9)		543	20%	
Obese (> 30)		516	19%	
Unknown		468	18%	
Median	24.7			
Mean	26.2			

### Incident pregnancies

**Fig. 1:**
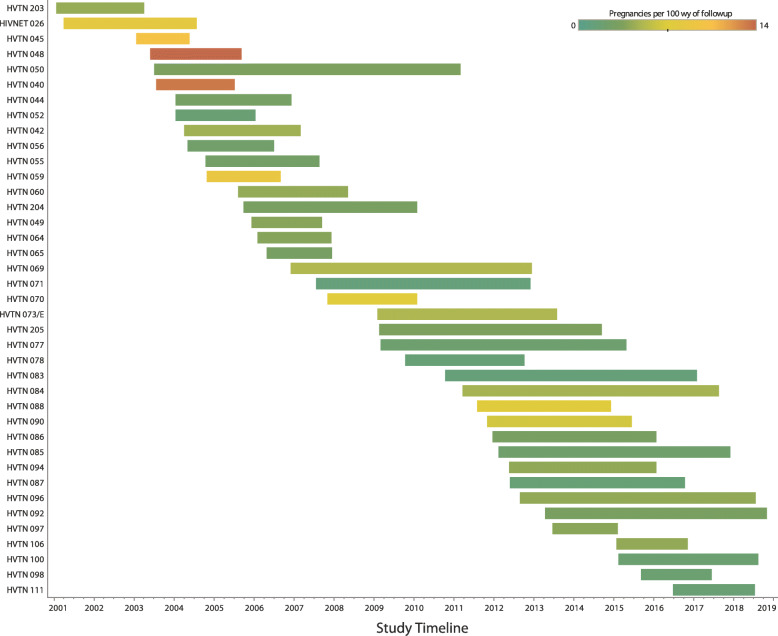
Pregnancy Rates by HIV Vaccine Trials Network study from 2001 to 2019

Overall, 193 pregnancies occurring in 172 individual participants were reported (Table 1). Most participants (153/193) had one pregnancy reported, while 17 participants had two pregnancies reported, and two participants had three reported pregnancies. Thirty-nine of the 53 studies included (74%) had at least one participant become pregnant during the study period. The overall pregnancy rate was 3.15 per 100 woman-years (w-yr) of follow-up, with a trend toward decreasing pregnancy rates during more recent studies (an average of 4.03 per 100 w-yr of follow-up from 2001 to 2006 compared with an average of 2.10 per 100 w-yr of follow-up from 2013 to 2018) (Fig. 1). As more Phase 1 and Phase 2a vaccine studies were conducted in North America (specifically the United States), more pregnancies occurred there than in other geographic regions. However, the pregnancy rate in North America was lower than the overall rate at 2.45 per 100 w-yr. Other geographic regions showed varying rates as well: Asia (Thailand) had the lowest rate at 1.56 per 100 2-yr, while remaining regions showed rates above the overall rate: Africa (Botswana, Malawi, South Africa, Tanzania, Zambia) at 3.35, Europe (Switzerland) at 4.11, South America (Brazil, Peru) at 6.07, and the Caribbean (Dominican Republic, Haïti, Jamaica, Puerto Rico, Trinidad and Tobago) at 8.53 pregnancies per 100 w-yr.

Most pregnancies occurred after all vaccinations had been completed (159/193, 82%), although 34/193 (18%) of pregnancies occurred prior to completion of the planned vaccine regimen and therefore led to early termination of vaccination. The pregnancy rate during main study participation was 3.09 per 100 woman-years. As pregnancy was no longer discouraged after the main study phase was completed, pregnancies occurred at a slightly greater rate in the LTFU phase (3.22 per 100 w-yr; Table 1).

### Pregnancy-related adverse events

We reviewed the dataset to determine if pregnancy-related adverse events (AEs) occurred at higher frequency in vaccinees vs placebo recipients. Amongst the 193 pregnancies reported following enrollment in HVTN studies through December 31, 2018, there were 22 maternal or fetal AEs of moderate or greater severity reported, all deemed unrelated to study product (Table 3). 11% of women who received at least one study product administration experienced a moderate or greater AE themselves or in their offspring, and 12% of women who received placebo experienced a moderate or greater AE themselves or in their offspring. Three congenital anomalies were reported among all pregnancy outcomes; these included one case of hepatic mesenchymal hamartoma, one case of Poland’s syndrome and one unspecified anomaly (Table 3). There was one life-threatening AE (HIV-1 infection in the study participant) and one maternal death (shock following an elective abortion).

**Table 3:** Perinatal Adverse Events

	Adverse Fetal Events		Adverse Maternal Events	
	Severity	Event	Severity	Event
Product recipients (*n* = 154 pregnancies)	Severe	Congenital anomaly in offspring	Death	Shock
	Severe	Congenital anomaly in offspring (Poland’s syndrome)	Life Threatening	HIV infected
	Severe	Hepatic Mesenchymal Hamartoma in Offspring	Severe	Pelvic Pain
			Severe	Placenta previa
			Moderate	Back Pain r/t herniated disk
			Moderate	Gastritis
			Moderate	Headache
			Moderate	Infected wisdom teeth - upper left + lower left
			Moderate	Insomnia
			Moderate	Left chest pains
			Moderate	Proteinuria
			Moderate	Sinusitis
			Moderate	Vaginal colonization with Group B strep
			Moderate	Wisdom teeth impaction - upper left + lower left
Placebo recipients (*n* = 39 pregnancies)			Severe	Incomplete abortion
			Severe	Increase interval (432 ms) from baseline QTc interval
			Moderate	Macro hematuria post therapeutic abortion
			Moderate	Pre-eclampsia
			Moderate	Retained placenta (with postpartum pyrexia)

### Pregnancy outcomes

Of the 193 pregnancies, 18 (9%) had unknown outcomes and were not included in the analysis of outcomes (13 amongst active vaccine recipients). Of the 175 pregnancies with known outcome, 32 resulted in an elective/therapeutic abortion (18%). Of the 143 pregnancies not terminated, 105 had full-term live births (73%) and adverse pregnancy outcomes were reported in 38 (27%). The adverse outcomes included 26 spontaneous abortion (< 20 weeks), 9 premature births (5.1% of known outcomes), and 3 spontaneous fetal demise and/or stillbirth (≥ 20 weeks), corresponding to a rate of 25.6 stillbirths per 1000 outcomes.

**Fig. 2:**
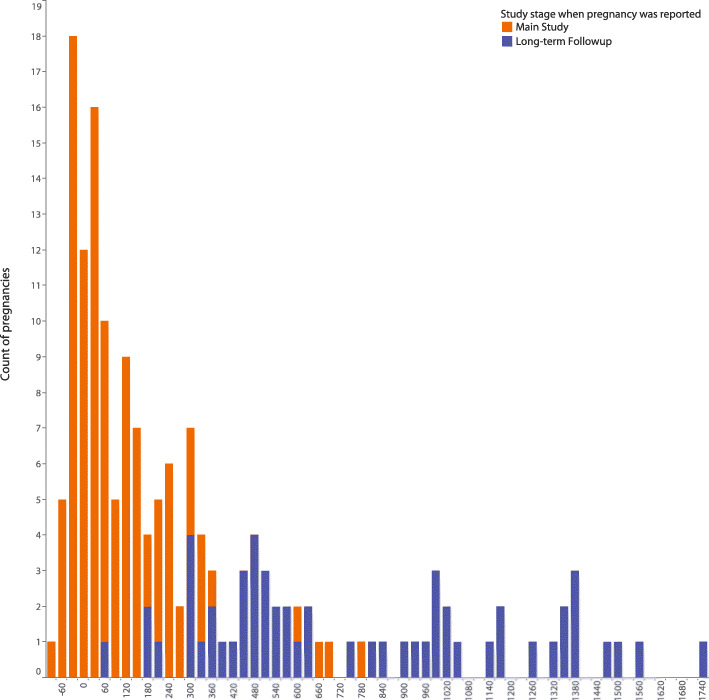
Number of Pregnancies per HVTN study

**Fig. 3:**
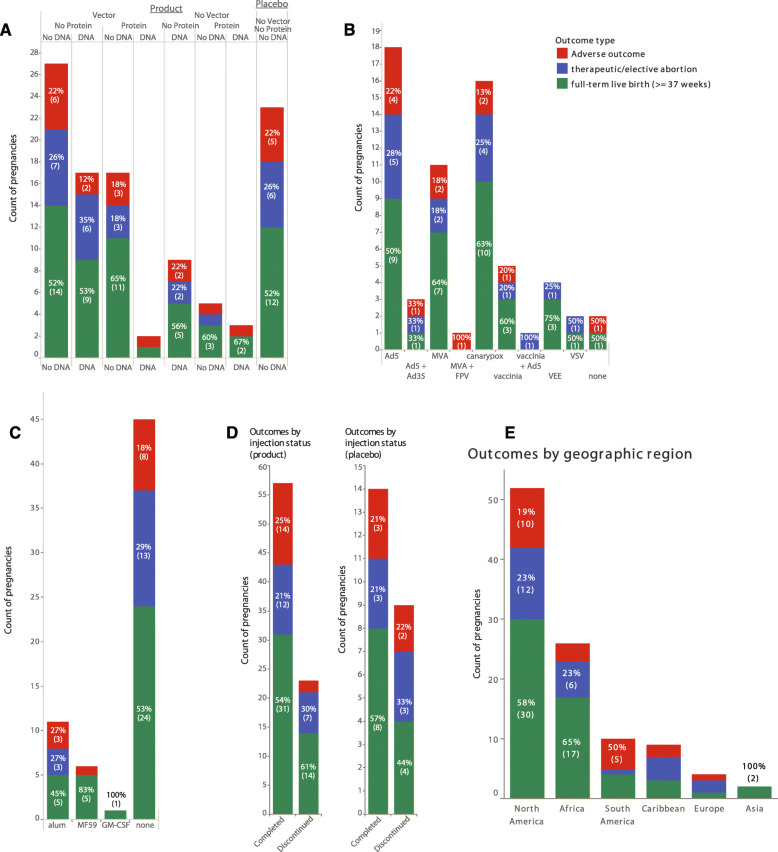
Pregnancy Outcomes in HVTN studies that occurred within 1 year of enrollment

For our primary analysis of pregnancy outcomes, we restricted our analysis to pregnancies which occurred within 1 year of the last product administration as timed by reported last menstrual period (LMP) (Fig. 2). This set included 111 pregnancies occurring in 106 study participants. Figure 3A shows that adverse pregnancy outcomes occur at similar rates amongst vaccine recipients and placebo recipients, and these rates were not statistically different from each other (*P* = 0.90). This lack of signal held true for viral vectored vaccines (Fig. 3B) as well as for adjuvanted protein vaccines (Fig. 3C) (*P* = 1.00 and *P* = 0.71, respectively). Additionally, when participants were divided into groups based on whether their vaccination series was stopped due to the identification of the pregnancy, no difference was seen in rates of adverse pregnancy outcomes in those whose vaccinations were stopped versus those who completed the vaccinations prior to pregnancy onset, (Fig. 3D) (*P* = 0.73). We also did not observe a difference in rates of adverse pregnancy outcomes between geographical regions (Fig. 3E) (*P* = 0.25). Further comparisons between outcome sub-categories, including adverse outcomes vs therapeutic/elective abortions, adverse outcomes vs live births, and therapeutic/elective abortions vs live births, revealed no statistically significant differences in any of the comparison groups (Additional Table 2).

We subsequently included pregnancies that occurred after 1 year following the final vaccination in an analysis of all pregnancies. Additional Fig. 1 demonstrates a similar absence of a safety finding when all pregnancies are examined, with no clear trend amongst categories of vaccine, vaccine components, by phase of the study, nor by geographic region.

Detailed data on all 193 pregnancies is available as Additional Table 3.

## Discussion

Increasing attention is being paid to the development of vaccines for adolescents prior to initiation of sexual activity to prevent incident infection and to the vaccination of pregnant women to confer protection to the newborn. Vaccine safety, particularly during pregnancy, is of considerable interest to the field [15–17], and we anticipate this analysis will provide valuable information for future HIV vaccine trials. Eliminating vertical transmission of HIV-1 is a high priority for prevention research and since young women and adolescent girls are likely to be a target for deployment of an effective HIV vaccine once one is developed, vaccine safety with respect to pregnancy will need to be established.

Our analysis revealed that 18% of conceptions among vaccine recipients occurred during the main study phase, though the overall pregnancy rate during study conduct was low (3.09 per 100 w-yr). A similar finding was described in the groundbreaking RV 144 trail in Thailand [18] where 1922 incident pregnancies were recorded in 6334 women (30%) in equal proportions among vaccine and placebo recipients, 362 of those (18%) occurring within the first 6 months of participation [19]. Not surprisingly, younger women aged 25 years and below contributed over 70% of the pregnancies. Conception was also more frequent after the vaccination period in the 352 South African women of reproductive potential enrolled in the HVTN 503 trial [20]; the overall incident pregnancy rate was 9.6/100 w-yr but 6.8/100 w-yr during the vaccination period vs 11.3/100 w-yr thereafter [11]. In HVTN 503, women agreed to avoid pregnancy for “one month after last vaccination”. After vaccinations were stopped for safety concerns, all pregnancies occurring within 6 months after enrollment were considered to be within the “vaccination period” even in those women who had received only a single vaccination, and had delayed conception for the subsequent 6 months [11]. The studies included in our analysis that took place during the same time period as the HVTN 503 trial recorded an average incident pregnancy rate of 4.6/100 w-yr, which was higher than the 2.3/100 w-yr recorded in subsequent trials and may in part be due to increasing availability of contraception options and, at least at some sites, a more structured approach to providing on-site effective contraception during this time. Indeed, HIV prevention researchers have actively sought correlates of incident pregnancy to identify potential participants with higher pregnancy risk prior to study entry and exclude them for reasons of trial efficiency and due to the lack of pre-clinical safety data in pregnancy [10, 20–22]. Increasing attention is being given to addressing vaccine safety during pregnancy and the ethics of conducting vaccine research among pregnant women [8, 15, 16, 23], including the rights and needs of high-risk populations who generally form the study population for efficacy trials of novel HIV prevention products [24]. Our findings contribute to the evidence base for such deliberations.

The Global Alignment of Immunization safety Assessment in pregnancy (GAIA) consortium [25] is one of the initiatives working to standardize tools for monitoring the safety of immunization programs in pregnancy, focusing on low- and middle-income countries. Another is the Brighton Collaboration [26], whose efforts to systematically collect potentially informative data internationally facilitate detection of rare events to improve global vaccine safety. To allow a more thorough analysis of pregnancy outcomes in terms of infant health along these guidelines, we propose to augment the information currently being collected in HIV-1 vaccine trials by adding the following variables to routinely-collected pregnancy outcome datasets: precise gestational age estimates and infant birth weight [27]. For vaccines nearing licensure, a pregnancy exposure registry could be established to collect pregnancy outcome data and possibly additional data on infants born to women vaccinated during pregnancy [28]; several licensed vaccines have such registries that could be used as models. Amongst other variables that would be of potential interest, more longitudinal infant data, including infant feeding method, immunological response to routine childhood vaccinations, anthropometric growth parameters, neurodevelopment and adverse events (including death) through age 6 months could be collected, potentially in post-licensure studies.

Our analyses provide a descriptive overview of the safety of prior HIV vaccines in pregnancy and provide reassuring data for estimating pregnancy safety in the large HVTN efficacy trials which are currently ongoing. Taken together, our data do not support an increased frequency of adverse pregnancy outcomes or pregnancy-related AEs in participants who have previously received experimental HIV-1 vaccines.

However, our study has limitations. Due to the diversity of the study populations across all included protocols, it is difficult to provide a comparator population for relative frequencies of pregnancies or adverse events to what is seen outside of the clinical trial context. The vaccine study products used were quite diverse and the number of pregnancies in recipients of any specific product was quite low. All participants who were capable of becoming pregnant were counselled to avoid pregnancy, contraception methods were reviewed throughout the trial periods, and negative pregnancy tests were confirmed prior to study product administration on the day of all vaccinations. Most pregnancies occurred after product administration had been completed, with many occurring over a year following last vaccination. Therefore, these data do not definitively establish that any of the products studied in HVTN trials are safe to administer during pregnancy.

However, these data do provide an estimated safety event rate which can inform sample size calculations for any future HIV-1 vaccine studies which specifically recruit pregnant women or participants who are considering pregnancy in the near future. As has been argued in the context of COVID-19 [29–31], when pregnant women and their infants are at specific risk of infectious diseases, earlier participation in clinical trials can be done ethically with an emphasis on the informed consent process. An important component of this will be increasing involvement of pregnant women and advocates on the community advisory boards to strengthen their involvement in designing and implementing HIV vaccine and prevention studies [32, 33]. Our study also identified additional variables for which data should be collected in future vaccine studies.

## Conclusions

We did not identify any apparent indications of increased risk of adverse pregnancy or birth outcomes in the early phase HIV-1 vaccine studies we analyzed. As there was considerable heterogeneity amongst the different vaccine trials, we suggest that more complete data on pregnancy outcomes should be collected in early phase HIV-1 vaccine clinical trials to better inform eventual efficacy trials.

## Supplementary Information

**Additional file 1: Fig. S1**. Pregnancy outcomes in HVTN vaccine studies that occurred at any time following enrollment.

**Additional file 2: Table S1**. Details of Study Products Received.

**Additional file 3: Table S2**. Comparison of Pregnancy Outcomes.

**Additional file 4: Table S3**. Pregnancy Detail Table**.**

## Data Availability

All data generated or analysed during this study are available as follows: A detailed dataset containing information on individual pregnancies, including vaccinations received by the pregnant individual and pregnancy outcomes is included in this published article and its supplementary information files. Additional datasets are available from the corresponding author on reasonable request.

## Abbreviations

AE: Adverse event
BMI: Body mass index
CRF: Case report form
DNA: Deoxyribonucleic acid
GAIA: Global alignment of immunization safety assessment in pregnancy
HIV-1: Human immunodeficiency virus type 1
HVTN: HIV vaccine trials network
LMP: Last menstrual period
LTFU: Long-term follow-up
NIAID: National institute of allergy and infectious diseases
NIH: National institutes of health
UNAIDS: Joint United Nations programme on HIV and AIDS
w-yr: Woman years
