# Supplementary material for: A retrospective analysis of incident pregnancy in phase 1 and 2a HIV-1 vaccine study participants does not support concern for adverse pregnancy or birth outcomes
Source: BMC Infect Dis. 2021 Aug 11;21:802. doi: 10.1186/s12879-021-06431-x (PMC8356543; doi:10.1186/s12879-021-06431-x)
Supplement: Supplementary file 1 — Additional file 1: Fig. S1. Pregnancy outcomes in HVTN vaccine studies that occurred at any time following enrollment. [file 12879_2021_6431_MOESM1_ESM.zip › Additional Figure 1.pdf]

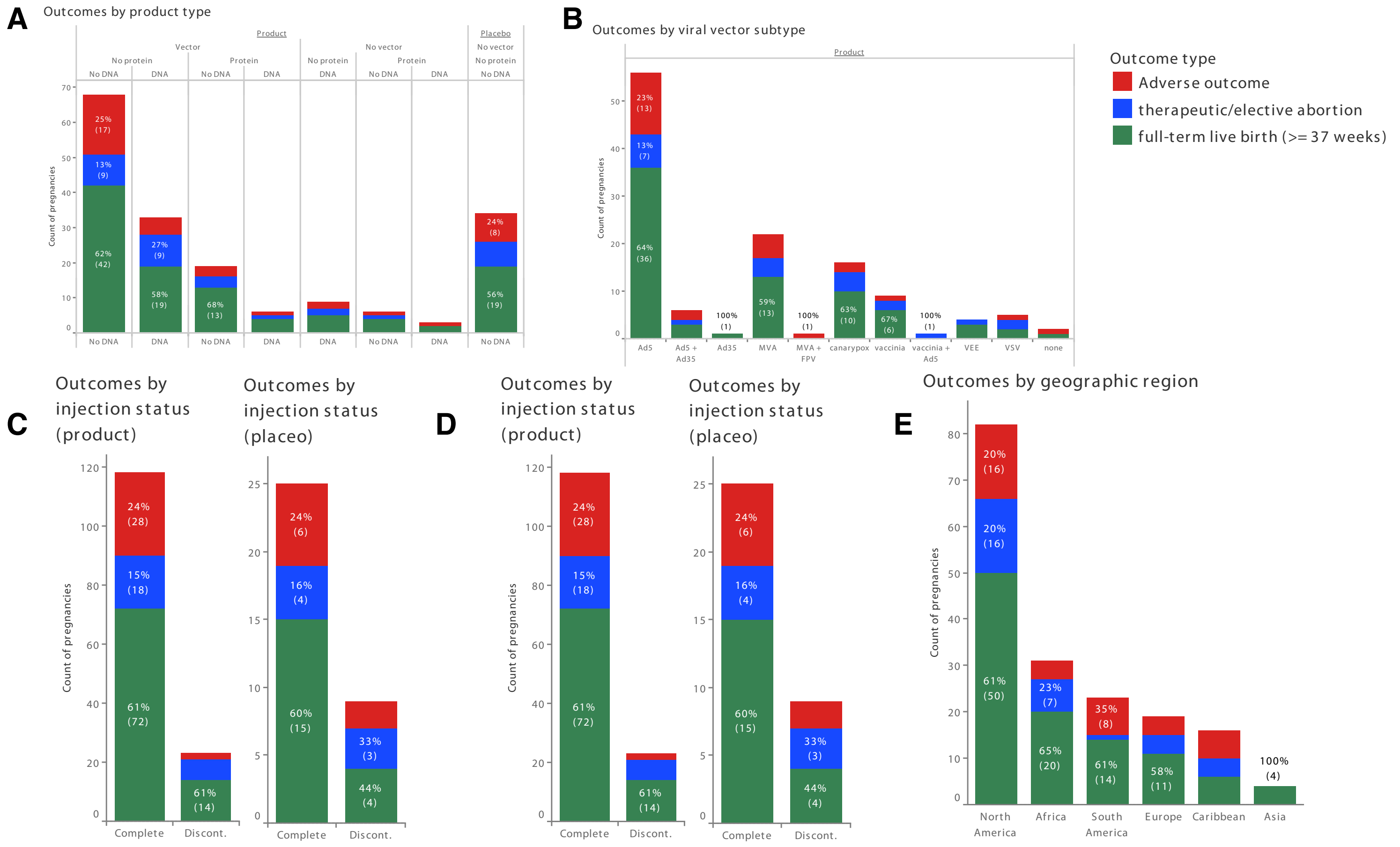

Additional Figure 1. Pregnancy outcomes in HVTN vaccine studies that occurred at any time following enrollment. Panel A shows outcomes of pregnancies by type of vaccine. Panel B shows outcomes of pregnancies stratified by the type of viral vector used. Panel C shows outcomes of pregnancies stratified by adjuvant used with protein vaccination. Panel D shows outcomes of pregancies stratified by whether injections had been completed or led to early discontinuation. Panel E shows outcomes stratified by geographical region.
