# Supplementary material for: A retrospective analysis of incident pregnancy in phase 1 and 2a HIV-1 vaccine study participants does not support concern for adverse pregnancy or birth outcomes
Source: BMC Infect Dis. 2021 Aug 11;21:802. doi: 10.1186/s12879-021-06431-x (PMC8356543; doi:10.1186/s12879-021-06431-x)
Supplement: Supplementary file 2 — Additional file 2: Table S1. Details of Study Products Received. [file 12879_2021_6431_MOESM2_ESM.pdf]

Additional Table 1. Details of study products received

Study	Study Treatment	Treatment Arm detail	N women of reproductive potential	Pregnancies in active phase	Pregnancies in Long-term followup
All	pooled controls	pooled controls	496	28	12
HIVNET 026	canarypox + protein	canarypox + gp120 (alum)	23	4	0
		canarypox + placebo	21	2	0
HVTN 039	canarypox	study arms with no pregnancies	26	0	0
HVTN 040	VEE	study arms with no pregnancies	6	0	0
		VEE (10^4 IU)	9	1	0
HVTN 041	protein	study arms with no pregnancies	17	0	0
HVTN 042	canarypox + lipopeptide	500 mcg lipopeptide	11	1	0
		canarypox	13	1	0
		canarypox + 150 mcg lipopeptide	11	1	0
		canarypox + 50 mcg lipopeptide	12	1	0
		study arms with no pregnancies	14	0	0
HVTN 044	DNA	DNA + 1.5 mg IL-2	7	1	0
		study arms with no pregnancies	10	0	0
HVTN 045	DNA	0.3 mg/mL DNA	7	1	0
		3.0 mg/mL DNA	10	1	0
HVTN 048	DNA	2.0 mg DNA	3	1	0
		4.0 mg DNA	4	1	0
		study arms with no pregnancies	5	0	0
HVTN 049	DNA + protein	100 mcg gp140 (MF59)	11	1	0
		250 mcg DNA + 100 mcg gp140 (MF59)	5	1	0
		study arms with no pregnancies	24	0	0
HVTN 050	Ad5	Ad5 (10^{10} vp)	63	2	5
		Ad5 (10^9 vp)	74	1	12
HVTN 052	DNA	study arms with no pregnancies	49	0	0
HVTN 054	Ad5	study arms with no pregnancies	15	0	0
HVTN 055	FPV + MVA	MVA (10^9 pfu/mL)	18	1	0
		study arms with no pregnancies	44	0	0
HVTN 056	peptide	peptide (GM-CSF)/RC529-SE	12	1	0
		study arms with no pregnancies	19	0	0
HVTN 057	DNA + Ad5 boost	study arms with no pregnancies	25	0	0
HVTN 059	VEE	study arms with no pregnancies	10	0	0
		VEE (10^5 IU)	10	1	0
		VEE (10^6 IU)	7	1	0
		VEE (10^8 IU)	5	1	0
HVTN 060	DNA + IL12 DNA + peptide	DNA 1500mcg + 1500 mcg IL-12 DNA (3 dose) + peptide (GM-CSF)	8	1	0
		DNA 1500mcg + 1500 mcg IL-12 DNA (5 dose)	15	1	0
		study arms with no pregnancies	32	0	0
HVTN 063	DNA	study arms with no pregnancies	6	0	0
	DNA + IL15 DNA	study arms with no pregnancies	24	0	0

Additional Table 1. Details of study products received

Study	Study Treatment	Treatment Arm detail	N women of reproductive potential	Pregnancies in active phase	Pregnancies in Long-term followup
	DNA + IL15 DNA + IL12 DNA	study arms with no pregnancies	14	0	0
HVTN 064	peptide	peptide (0.2 mg)(alum)	4	1	0
		study arms with no pregnancies	21	0	0
HVTN 065	DNA + MVA	3 mg DNA (1 dose) + MVA (10^8 $TCID_{50}$) (2 dose)	16	1	0
		study arms with no pregnancies	41	0	0
HVTN 067	DNA + MVA	study arms with no pregnancies	13	0	0
HVTN 068	Ad5	study arms with no pregnancies	14	0	0
	DNA + Ad5	study arms with no pregnancies	13	0	0
HVTN 069	DNA + Ad5	4 mg DNA ID + Ad5 (10^{10} PU)	14	0	4
		4 mg DNA IM + Ad5 (10^{10} PU)	13	0	1
		4 mg DNA SC + Ad5 (10^{10} PU)	10	1	0
HVTN 070	DNA + IL12 DNA	6 mg DNA + 1.5 mg IL-12 DNA	13	3	0
		6 mg DNA + 2 mg IL-15 DNA	13	1	0
		study arms with no pregnancies	20	0	0
HVTN 071	Ad5	Ad5 (1.5 x 10^{10} Ad vg)	21	1	0
HVTN 072	DNA + Ad5 + Ad35	study arms with no pregnancies	4	0	0
HVTN 073/E	DNA + MVA + protein	4 mg DNA + MVA (1.49 x 10^9 pfu)	10	1	0
		4 mg DNA + MVA (1.49 x 10^9 pfu) + 100 mcg gp140 (MF59)	7	2	0
		study arms with no pregnancies	3	0	0
HVTN 076	DNA + Ad5	study arms with no pregnancies	6	0	0
HVTN 077	DNA + Ad5 + Ad35	4 mg DNA + Ad35 (10^{10} PU)	17	0	1
		Ad5 (10^{10} PU) + Ad35 (10^{10} PU)	19	1	4
		study arms with no pregnancies	34	0	0
HVTN 078	attenuated vaccinia + Ad5	study arms with no pregnancies	32	0	0
		vaccinia (10^7 PFU) + Ad5 (10^{10} PU)	22	1	0
HVTN 080	DNA + IL12 DNA	study arms with no pregnancies	21	0	0
HVTN 082	DNA + Ad5	study arms with no pregnancies	8	0	0
HVTN 083	Ad5	Ad35 (10^{10} PU) + Ad5 (Env A) (10^{10} PU)	18	0	1
		Ad35 (10^{10} PU) + Ad5 (Env B) (10^{10} PU)	9	1	0
		Ad5 (Env A) (10^{10} PU)	23	0	1
		Ad5 (Env A) (10^{10} PU) + Ad5 (Env B) (10^{10} PU)	18	0	2
		study arms with no pregnancies	10	0	0
HVTN 084	Ad5	Ad5 (Env A/B/C) (10^{10} PU)	43	0	8
		Ad5 Gag-Pol (5 x 10^9 PU)	39	0	11
HVTN 085	Ad5	Ad5 Gag-Pol (5 x 10^{10} PU)	10	0	2

Additional Table 1. Details of study products received

Study	Study Treatment	Treatment Arm detail	N women of reproductive potential	Pregnancies in active phase	Pregnancies in Long-term followup
		Ad5 Gag-Pol/Env A/B/C (0.25 x 10^{10} PU)	16	0	2
		study arms with no pregnancies	11	0	0
HVTN 086	DNA + MVA + protein	4 mg DNA + 100 mcg gp140 (MF59)	20	0	1
		4 mg DNA + MVA (1.45 x 10^9 pfu)	17	1	1
		MVA (1.45 x 10^9 pfu) / 100 mcg gp140 (MF59)(coadministered)	18	1	1
		MVA (1.45 x 10^9 pfu) + 100 mcg gp140 (MF59)	21	0	2
HVTN 087	DNA + IL12 DNA + VSV	3 mg DNA + 1500 mcg IL-12 DNA + VSV (3.4 x 10^7 PFU)	5	0	1
		study arms with no pregnancies	25	0	0
HVTN 088	protein	100 mcg gp140 (MF59)	5	1	0
HVTN 090		Previously vaccinated + 100 mcg gp140 (MF59)	8	1	0
	VSV	study arms with no pregnancies	4	0	0
		VSV (10^4 PFU)	6	0	2
		VSV (10^5 PFU)	5	0	1
		VSV (10^7 PFU)	4	0	1
		VSV (10^8 PFU)	4	0	1
HVTN 092	DNA + attenuated vaccinia	4 mg DNA (3 doses over 1 month) + vaccinia (1.2 x 10^8 PFU)	27	1	0
		4 mg DNA (3 doses over 2 months) + vaccinia (1.2 x 10^8 PFU)	15	1	0
		study arms with no pregnancies	25	0	0
HVTN 094	DNA + MVA	0.3mg DNA + MVA (10^8 $TCID_{50}$) (over 10 months)	14	0	1
		0.3mg DNA + MVA (10^8 $TCID_{50}$) (over 8 months)	11	1	1
HVTN 096	DNA + attenuated vaccinia + protein	4 mg DNA (2 doses) + vaccinia (15 x 10^6 PFU)(2 doses)	10	0	1
		4 mg DNA (2 doses) + vaccinia (15 x 10^6 PFU)(2 doses) + 600 mcg gp120 (2 doses)(alum)	11	0	4
		vaccinia (15 x 10^6 PFU) (4 doses) + 600 mcg gp120 (4 doses)(alum)	10	2	0
		vaccinia (15 x 10^6 PFU)(4 doses) + 600 mcg gp120 (2 doses)(alum)	10	1	0
HVTN 097	canarypox + protein	canarypox (10^6 $CCID_{50}$) + gp120 (600 mcg)(MF59)	9	1	0
		Tetanus toxoid (40 I.U./0.5 mL) + canarypox (10^6 $CCID_{50}$) + gp120 (600 mcg)(MF59) + HBV (20mcg)	29	1	0

Additional Table 1. Details of study products received

Study	Study Treatment	Treatment Arm detail	N women of reproductive potential	Pregnancies in active phase	Pregnancies in Long-term followup
HVTN 098	DNA + IL12 DNA	study arms with no pregnancies	35	0	0
HVTN 100	canarypox + protein	canarypox (10^6 $CCID_{50}$) + gp120 (200 mcg)(MF59)	88	3	0
HVTN 105	DNA + protein	study arms with no pregnancies	48	0	0
HVTN 106	DNA + MVA	4 mg DNA (clade B) + MVA (10^8 PFU)	11	1	0
		4 mg DNA (consensus) + MVA (10^8 PFU)	11	1	0
		study arms with no pregnancies	15	0	0
HVTN 110	Ad4 + protein	study arms with no pregnancies	4	0	0
HVTN 111	DNA + protein	4 mg DNA (2 doses via Biojector) + 200 mcg gp120 (3 doses)(MF59)	11	1	0
		study arms with no pregnancies	50	0	0
HVTN 112	DNA + VSV	study arms with no pregnancies	4	0	0
HVTN 203	canarypox + protein	canarypox ($10^{7.26}$ $TCID_{50}$) + 600 mcg gp120 (2 doses)(alum)	19	1	0
		canarypox ($10^{7.26}$ $TCID_{50}$) + 600 mcg gp120 (3 doses)(alum)	15	4	0
		study arms with no pregnancies	27	0	0
HVTN 204	DNA + Ad5	4 mg DNA + Ad5 (10^{10} PU)	127	7	0
HVTN 205	DNA + MVA	3 mg DNA + MVA (10^8 $TCID_{50}$)	67	2	4
		MVA (10^8 $TCID_{50}$)	27	1	2
**Total**			**2673**	**103**	**90**
