# Supplementary material for: A retrospective analysis of incident pregnancy in phase 1 and 2a HIV-1 vaccine study participants does not support concern for adverse pregnancy or birth outcomes
Source: BMC Infect Dis. 2021 Aug 11;21:802. doi: 10.1186/s12879-021-06431-x (PMC8356543; doi:10.1186/s12879-021-06431-x)
Supplement: Supplementary file 3 — Additional file 3: Table S2. Comparison of Pregnancy Outcomes. [file 12879_2021_6431_MOESM3_ESM.pdf]

**Additional Table 2.** Comparison of Pregnancy Outcomes amongst Different Groups

| Comparisons | Fisher's Exact Test P-value | FDR q-value |
|---|---|---|
| Outcomes by Adjuvants | | |
| Adverse Outcome | 0.8904 | 0.8904 |
| Full-Term Live Birth | 0.3781 | 0.8904 |
| Therapeutic/Elective Abortion | 0.6253 | 0.8904 |
| Outcomes by Geographic Region | | |
| Adverse Outcome | 0.2013 | 0.3607 |
| Full-Term Live Birth | 0.2404 | 0.3607 |
| Therapeutic/Elective Abortion | 0.4021 | 0.4021 |
| Outcomes by Injection Status | | |
| Adverse Outcome | 0.4506 | 0.8544 |
| Full-Term Live Birth | 0.8544 | 0.8544 |
| Therapeutic/Elective Abortion | 0.6763 | 0.8544 |
| Outcomes by Product Types | | |
| Adverse Outcome | 0.8631 | 0.9926 |
| Full-Term Live Birth | 0.9926 | 0.9926 |
| Therapeutic/Elective Abortion | 0.9569 | 0.9926 |
| Outcomes by Treatments (Vaccine vs Placebo) | | |
| Adverse Outcome | 1 | 1 |
| Full-Term Live Birth | 0.8135 | 1 |
| Therapeutic/Elective Abortion | 0.789 | 1 |
